# Stellate ganglion block causing reproducible improvement of allergic rhinitis and asthma: a case report

**DOI:** 10.1097/PR9.0000000000001431

**Published:** 2026-04-07

**Authors:** Azeem Ahmad, Efrain Perez-Bravo

**Affiliations:** ABBEL Research Division, Rehabilitation Institute at Sinai (formerly Sinai Rehabilitation Center), Sinai Hospital of Baltimore, Baltimore, MD, USA

**Keywords:** Autonomic dysfunction, Validated outcome measures, Interventional pain, Sympathetic blockade, Atopic airway disease

## Abstract

**Introduction:**

Stellate ganglion block (SGB) is hypothesized to influence immunologic and physiologic processes through autonomic modulation. Though similar effects of SGB on atopic disease have been intermittently observed over the past century, methodological concerns limit the internal and external validity of these prior efforts.

**Objectives:**

To describe a case of simultaneous and reproducible improvement in allergic rhinitis and asthma after SGB, and to contextualize this observation within the existing literature.

**Methods:**

A 35-year-old woman with Complex Regional Pain Syndrome (CRPS) following a radial head fracture underwent SGB as a part of routine CRPS management. Her incidentally-observed changes in allergic rhintis and asthma were assessed longitudinally using validated clinical outcome measures in a retrospective fashion.

**Results:**

The patient demonstrated improvement of her allergic rhinitis and asthma after SGB, exceeding MCID thresholds for meaningful clinical improvement of each condition. These benefits persisted for 9 months before a partial recurrence of her atopic airway disease. A second SGB was able to reproduce these improvements, again exceeding MCID thresholds for both allergic rhinitis and asthma.

**Conclusions:**

SGB's effects on atopic disease warrant reappraisal of SGB as a potential adjunct for atopic airway disease, particularly in light of recent experimental evidence suggesting that SGB may exert an immunomodulatory effect. This case therefore highlights a meaningful gap in the literature and underscores the need for systematic investigation of SGB as a potential adjunctive treatment for atopic airway disease.

## Abstract 2

Supplemental Digital Content is Available in the Text.

This case report quantifies the effect of stellate ganglion blockade on atopic airway disease, with a mechanistic review highlighting this as a potential immunomodulatory adjunct.

## 1. Introduction

Allergic rhinitis and asthma are inflammatory diseases of the upper and lower airway, respectively, characterized by variable obstruction, hypersensitivity to irritants, and substantial impact on quality of life. Modern treatment has shifted from autonomic-based symptom control to immunomodulatory disease-modification, improving patient outcomes.^5^ However, severe or refractory cases remain problematic, in part due to the morbidity of third-line therapies such as systemic corticosteroids.^34^ Both conditions carry major economic burdens, with US costs estimated at $1.7 billion annually for allergic rhinitis and $81.9 billion for asthma.^28,30^ These burdens highlight the need for innovative therapies—and, perhaps, a reappraisal of old ideas.^2,34^

This case describes a patient with complex regional pain syndrome (CRPS) who experienced reproducible improvement of refractory allergic rhinitis and asthma after a stellate ganglion block (SGB). It “rediscovers” an association not explored in the Western world since the 1950s and improves upon more-recent but sporadic international research by using validated outcome measures. The accompanying literature review integrates recent preclinical evidence with these earlier reports, bringing this association into the modern era. Furthermore, the simultaneous improvement of allergic rhinitis and asthma in this case is unique, with meaningful implications for the “unified airway hypothesis” linking these conditions.^3,6^ This case thus highlights autonomic modulation as a potential adjunct for atopic airway disease.

## 2. Methods

### 2.1. Ethics and consent

This article follows CARE guidelines. Written informed consent was obtained for case report preparation, and an IRB exemption (#2025-036) was granted. SGBs were performed as per routine CRPS management.

### 2.2. Intervention details

After consent, each SGB was performed under fluoroscopy with the patient supine. A 25-gauge spinal needle was advanced under multiplanar guidance through the prevertebral fascia to the junction of the C6 tubercle and anterior vertebral body. After negative aspiration, 1 cc of contrast demonstrated appropriate subfascial spread along the longus colli in a narrow cranio-caudal band, confirming placement. This was followed by 1 cc of 0.25% ropivacaine as a test dose and 5 cc of ropivacaine for treatment. The patient reported ipsilateral sensory changes and improvement of her CRPS symptoms within minutes of each block, serving as further evidence of successful placement. The patient was observed postprocedure and discharged, with no complications noted at follow-up.

### 2.3. Data collection

Clinical data including atopic history were obtained through chart review and structured interviews. Chart review included clinical notes and medication refills. Interviews were conducted at 1 week and 4 months after the second SGB to capture rhinitis and asthma outcomes, alongside routine clinical follow-up.

### 2.4. Outcome measures

Clinical significance was determined using multiple standardized tools to maximize external validity. Asthma control was assessed with the Asthma Control Questionnaire-5 (ACQ-5; MCID = 0.5),^15^ the Asthma Control Test (ACT; MCID = 3),^32^ and the GINA Symptom Control Tool (categorical changes in lieu of MCID).^10^ Allergic rhinitis was per the Total Nasal Symptom Score (TNSS; MCID = 3.6)^27^ and Rhinitis Control Assessment Test (RCAT; MCID = 3).^26^ All questionnaires were clinician-administered.

### 2.5. Follow-up and analysis

**Figure 1.**
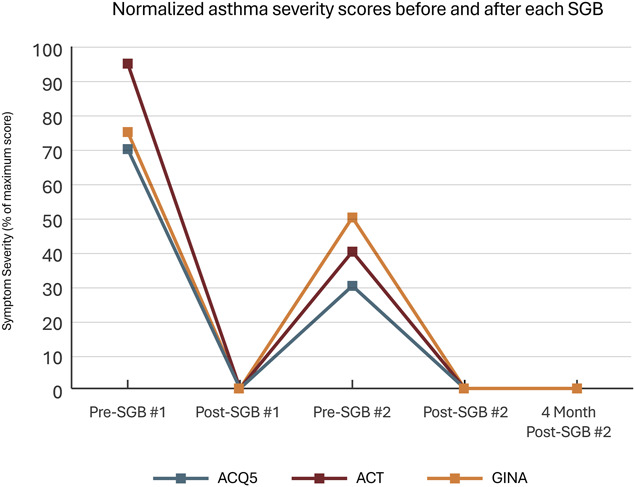
Graph showing trends of asthma severity after stellate ganglion block (SGB)—all 3 measures crossed the threshold for clinically significant change when moving between each timepoint, with exception being no significant change from post-SGB #2 to 4 months post-SGB #2 for all 3 measures; on this normalized graph, minimal clinically important difference (MCID) for asthma control questionnaire-5 = 0.5/6 = 8.3% and for asthma control questionnaire = 3/20 = 15%.

**Figure 2.**
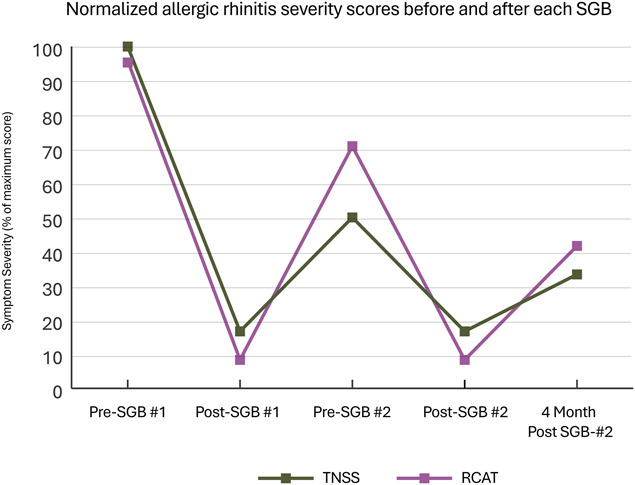
Graph showing trends of allergic rhinitis severity after stellate ganglion block (SGB)—both measures crossed the threshold for clinically significant change when moving between each timepoint with exception of total nasal symptom score not crossing significance when going from post-SGB #2 to 4 months post-SGB #2; note, time axis is not to scale; on this normalized graph, minimal clinically important difference (MCID) for total nasal symptom score = 3.6/12 = 30% and for rhinitis control assessment test = 3/24 = 12.5%.

Outcomes data were consolidated into 5 timepoints (Table 1, see Fig. S1, supplemental digital content, http://links.lww.com/PR9/A397). Changes exceeding the previously described MCID threshold were noted and summarized in Table 1. Figures 1 and 2 normalize each questionnaire against its maximum score to simplify visual comparison across measures.

**Table 1:** Patient symptom timeline across 5 timepoints.

Date	Clinical timepoint	GINA (0–4; higher = more severe)	ACT (5–25; higher = less severe)	ACQ5 (0–6; higher = more severe)	TNSS (0–12; higher = more severe)	RCAT (6–30; higher = less severe)
5/8/24	Pre-SGB #1	3 (uncontrolled)	6 (very poorly controlled)	4.2 (not well-controlled)	12	7
5/29/24	Post-SGB #1	0 (controlled)*	25 (well-controlled)*	0 (well-controlled)*	2*	28*
5/21/25	Pre-SGB #2	2 (partly controlled)*	17 (not well-controlled)*	1.8 (not well-controlled)*	6*	13*
6/4/25	Post-SGB #2	0 (controlled)*	25 (well-controlled)*	0 (well-controlled)*	2*	28*
9/15/25	4 mo post-SGB #2	0 (controlled)	25 (well-controlled)	0 (well-controlled)	4	20*

Pre-SGB indicates the patient's symptoms immediately before her SGB, and post-SGB indicates the patient's symptoms approximately 2 wk after her SGB.

* Where the questionnaire score exceeds the “clinical significance” threshold as compared with the prior value, these are defined as (ACQ-5; MCID = 0.5), (ACT; MCID = 3), (GINA; categorical changes), (TNSS; MCID = 3.6) and (RCAT; MCID = 3).

SGB, stellate ganglion block; TNSS, total nasal symptom score; MCID, minimal clinically important difference; RCAT, rhinitis control assessment test.

## 3. Results

### 3.1. Patient background and baseline atopic disease

A 35-year-old woman with fibromyalgia, PTSD, stable schizophrenia, allergic rhinitis, and asthma sustained a left radial head fracture in 2023, managed nonoperatively. Three months postinjury, allodynia, edema, and altered hair growth developed in the left arm, prompting referral to PM&R with concern for CRPS.

At baseline, rhinitis and asthma were poorly controlled despite trialing albuterol, budesonide/formoterol, cetirizine, montelukast, azelastine, and intranasal fluticasone. She had at least 1 ED presentation for bronchospasm within the past 5 years. Her baseline symptom severity is quantified under the Pre-SGB #1 timepoint in Table 1 and supplemental digital content (see Fig S1, http://links.lww.com/PR9/A397).

### 3.2. Complex regional pain syndrome outcomes

After excluding cervical radiculopathy with an MRI and confirming Budapest criteria, CRPS-1 was diagnosed with a baseline CRPS Severity Score (CSS) of 12 of 16. An SGB in May 2024 reduced her CSS to 4. Symptoms recurred 9 months later, with a repeat SGB in May 2025 reducing her CSS from 6 to 2.

### 3.3. Atopic outcomes

While preparing for the second SGB, the patient reported her rhinitis and asthma had incidentally improved after the first procedure. Structured interviews after the second SGB confirmed clinically significant improvements across all outcome measures (Table 1, Figs. 1 and 2). These improvements allowed her to resume employment and daily errands without limitation. Albuterol usage fell from >6 actuations/day to <1 per month.

At 4 months after her second SGB, her asthma remains subclinical. Mild rhinitis recurred, exceeding MCID for RCAT but not TNSS, but is manageable with montelukast and levocetirizine. She reports a typical pattern of her rhinitis being “completely gone” for approximately 2 to 4 weeks post-SGB, followed by gradual recurrence. She remains highly satisfied and requests repeat SGB if her symptoms relapse.

## 4. Discussion

This is the first article to document reproducible improvement of allergic rhinitis or asthma after SGB using validated instruments. It may seem paradoxical that blocking sympathetic outflow could improve either condition, as sympathetic agonists offer symptom control for both diseases, but compelling evidence underlies this phenomenon.

### 4.1. Proposed mechanisms of action

#### 4.1.1. Immunomodulatory mechanisms

Stellate ganglion block and stellate irradiation in preclinical models of asthma reduce airway inflammation by decreasing immune cell infiltration and Th2 cytokines (eg, IL-4, IL-5, IL-13).^8,42,43^ In human asthmatics, SGB reduces systemic histamines and leukotrienes in a dose-dependent fashion.^37^ Although fewer comparable studies exist for allergic rhinitis, available evidence suggests a benefit on overall symptom severity.^6,16,18,33^ Furthermore, SGB can temper the immune response in other disease states of interest.^7,12,20,22,40,44^

For atopic airway disease, this immunomodulatory effect likely arises through shared neural mediators governing the local immune environment.^9^ This common neurologic basis, the comorbidity of allergic rhinitis and asthma, and the interrelatedness of their symptom flares have led to the development of the “unified airway hypothesis” linking both conditions.^3,11^ It may be that SGB is able to produce its clinical effects by way of this shared hypothetical pathway—however, this has not yet been experimentally established.

#### 4.1.2. Psychophysiologic mechanisms

Stellate ganglion block may influence the subjective experience of airway disease. Stellate irradiation reduces dyspnea sensation during induced airflow restriction in healthy subjects, and SGB has been associated with improvement of PTSD and anxiety symptoms.^14,23^

Furthermore, SGB's effect on a patient's psychological state may directly influence their atopic symptoms. The bidirectional relationship between psychologic factors and atopic disease has long been recognized.^1,17,21,41^ This has even been described for SGB—one patient who had relapse of her asthma with emotional distress saw improvement with repeat SGB.^24^

Taken altogether, a psychological effect of SGB may contribute to a perceived or actual benefit on atopic disease but likely acts in conjunction with the other immunologic and physiologic changes described in this article.

#### 4.1.3. Direct neurogenic control of airway diameter

Evidence for SGB directly modulating neurogenic airway control is less robust. Nonasthmatics do not demonstrate a dose–response relationship in pulmonary function after SGB, but comparable studies are needed for asthmatics.^19^ SGB does reduce bronchial reactivity in animal models, but comparable studies showing decreased airway resistance for human asthmatics are methodologically limited.^8,37^

For allergic rhinitis, even less pertinent data exist. Patients with rhinitis and controls demonstrate *decreased* nasal patency immediately after SGB, consistent with successful sympathetic blockade, but no assessment has been made in patients with positive responses to SGB treatment.^13,41^

Beyond that, SGB reduces norepinephrine and cortisol levels; some argue that chronically elevated sympathetic hormone levels can exacerbate allergic rhinitis and asthma severity, but this remains controversial.^4,17,39,44^

Further physiologic research in humans is needed to clarify the mechanisms of SGB's effect on allergic rhinitis and asthma.

### 4.2. Limitations of this article

This case report has limitations. Questionnaires were administered retrospectively, thus recall bias is possible. Improvement in psychiatric comorbidities could confound these results, although the patient denied meaningful changes in her PTSD or schizophrenia symptoms. Pulmonary function testing could have corroborated the questionnaires with objective data; unfortunately, this was not feasible to arrange for this patient. Furthermore, no standard regimen for SGB has been established in this context; historical reports suggest multiple SGBs are often required.^16,25,29,31,33,35–38^ Finally, even with the advent of modern imaging guidance, updated safety data are needed on SGB including a comparison of fluoroscopic and ultrasound-guided techniques. Thus, it is clear more evidence is needed before SGB can be recommended as a routine adjunctive therapy in atopic disease.

## 5. Conclusions

This case links historical sympathetic interventions to modern management of atopic airway disease. Independent chains of evidence link SGB to allergic rhinitis and asthma; if the autonomic system is the clinical effector underlying the “unified airway” hypothesis, SGB has value both therapeutically and as a method to identify additional targets for immunomodulation. Given compelling preclinical data and limited modern clinical research on the topic, interdisciplinary collaboration between pain medicine, allergy, and pulmonary specialists is warranted to define the therapeutic and mechanistic role of stellate ganglion block in atopic disease.

## Disclosures

The authors have no conflicts of interest to declare.

## Supplemental digital content

Supplemental digital content associated with this article can be found online at http://links.lww.com/PR9/A397.

## Supplementary Material

SUPPLEMENTARY MATERIAL
