# Supplementary material for: Stellate ganglion block causing reproducible improvement of allergic rhinitis and asthma: a case report
Source: Pain Rep. 2026 Apr 7;11(3):e1431. doi: 10.1097/PR9.0000000000001431 (PMC13061519; doi:10.1097/PR9.0000000000001431)
Supplement: SUPPLEMENTARY MATERIAL [file painreports-11-e1431-s001.pdf]

Supplemental Figure S1

CLINICAL EVENT		CLINICAL DATAPOINT
Patient presents to the ED for “bronchospasm”	6/1/18	
Patient reports treatment failure on monteleukast, cetirizine, intranasal fluticasone, intranasal azelastine, and inhaled budesonide-formoterol for her atopic disease	1/15/19	
Patient falls, sustaining a L radial head fracture managed non-operatively	11/17/23	
OT notes allodynia, edema, and altered hair growth concerning for CRPS	2/28/24	
Patient establishes with PM&R clinic	3/18/24	
	5/8/24	**Pre-SGB #1 datapoint, assigned retrospectively**
SGB #1 is performed	5/15/24	
	5/29/24	**Post-SGB #1 datapoint, assigned retrospectively**
Patient's allergic rhinitis symptoms begin to become disruptive again	2/15/25	
Patient first reports her asthma “went away” after the first injection	4/29/25	
	5/15/25	**Pre-SGB #2 datapoint, assigned retrospectively**
SGB #2 is performed	5/21/25	
First structured interview with the patient, patient first reports that her allergic rhinitis symptoms "disappeared” with each SGB	5/29/25	
Patient seen in clinic and reiterates persistent relief of her symptoms	6/4/25	**Post-SGB #2 datapoint, assigned retrospectively**
Second structured interview with the patient	9/15/25	**4 Months Post-SGB #2 datapoint**

Figure Legend

- *Supplemental Figure S1: Timeline covering pertinent events in the patient's atopic disease history through the incident injury and subsequent CRPS diagnosis, with visual representation of when the post-SGB datapoints were assigned relative to the patient's overall clinical course*
